# Supplementary material for: DNA epigenetic marks are linked to embryo aberrations in amphipods
Source: Sci Rep. 2020 Jan 20;10:655. doi: 10.1038/s41598-020-57465-1 (PMC6971077; doi:10.1038/s41598-020-57465-1)
Supplement: Supplementary file 1 — Supplementary Information. [file 41598_2020_57465_MOESM1_ESM.pdf]

DNA epigenetic marks are linked to embryo aberrations in amphipods

Elena Gorokhova[1], Giulia Martella[1], Nisha H. Motwani[1], Natalia Y. Tretyakova[2], Brita Sundelin[1] and Hitesh V. Motwani[1,*]

[*]Corresponding author (hitesh.motwani@aces.su.se)

Supplementary Information

Contents *Page*

Note S1. Description of *Monoporeia affinis* eggs in embryo development stages 4–8. 3

Figure S1. Map showing locations in the Baltic region from where the amphipods used in the present study were collected within SNMMP. 4

Figure S2. Illustration of nucleoside adduct (M) and its fragmentation pattern. 5

Figure S3. Evaluation of the method for quantification of nucleoside adducts using calf thymus DNA (ctDNA). 6

Figure S4. Identification of 5-me-dC (A3), N^6-me-dA (A22) and 8-oxo-dG (A21) in amphipods DNA using respective standards. 7

Figure S5. Distribution of the measured nucleoside adducts (as well as dA, dC and T) shown as histograms and box-plots. 8

Figure S6. Variations in the normalized peak area for nucleoside adducts as well as dA, dC and T in *M. affinis* females and their embryos. 9

Figure S7. NMDS ordination diagram based on the nucleoside adducts in the females and their embryos with (A) healthy and (B) unhealthy animals. 10

Figure S8. Discrimination of the adductome in healthy and unhealthy amphipods based on area under the receiver operating characteristic (ROC) curve (AUC). 11

Table S1. Summary of embryo analysis in gravid females. 12

Table S2. Cross-correlation (Pearson r) for specific nucleoside adducts between the female and the embryo DNA. 13

Table S3. Pair-wise correlations (Pearson r) for specific nucleoside adducts within an individual (A) female and (B) embryo DNA. 14

Table S4. Logistic regression output. 15

Note S1. Description of *Monoporeia affinis* eggs in embryo development stages 4–8. (adapted from Sundelin and Eriksson, 1998)

Stage 4, length (*l*) 0.53–0.58 mm: Gastrulation begins from the posterior region of the germinal disc. The embryonic mesoderm and endoderm are formed due to ventrally gastrulation proceeding by inward proliferation of the cells. The outer egg membrane is burst and the developing embryo is allowed to increase in size. Later the caudal furrow is developed by formation of a narrow groove.

Stage 5, *l* 0.59–0.63 mm: The differentiation of appendages and embryo organs. After gastrulation, the embryo is characterized by its comma-like shape and the dorsal organ is formed.

Stage 6, *l* 0.66–0.69 mm: At this stage, the midgut extends backwards, cephalothorax is visible and the budlike limbs start to develop. The dorsal organ is at a maximum size.

Stage 7, *l* 0.70–0.73 mm: Clublike antennae of the cephalothorax develop and appendages are segmented and the first pigments of the eyespot appear.

Stage 8, *l* 0.77–0.81 mm: The dorsal organ has regressed and the compound eye is fully developed and clearly visible. Peristalsis begins in the midgut and there is movement in the limbs and body.

For representative pictures of embryonic development see Fig. 3 in Sundelin and Eriksson, 1998.

Figure S1. Map showing locations in the Baltic region from where the amphipods used in the present study were collected within SNMMP. Hydrographical factors in terms of bottom depth (20–40 m), sediment type (soft bottom), salinity (5–7 PSU), and bottom-layer temperature (~5–6 °C, all year round in all basins) were relatively constant at all stations[19,20].

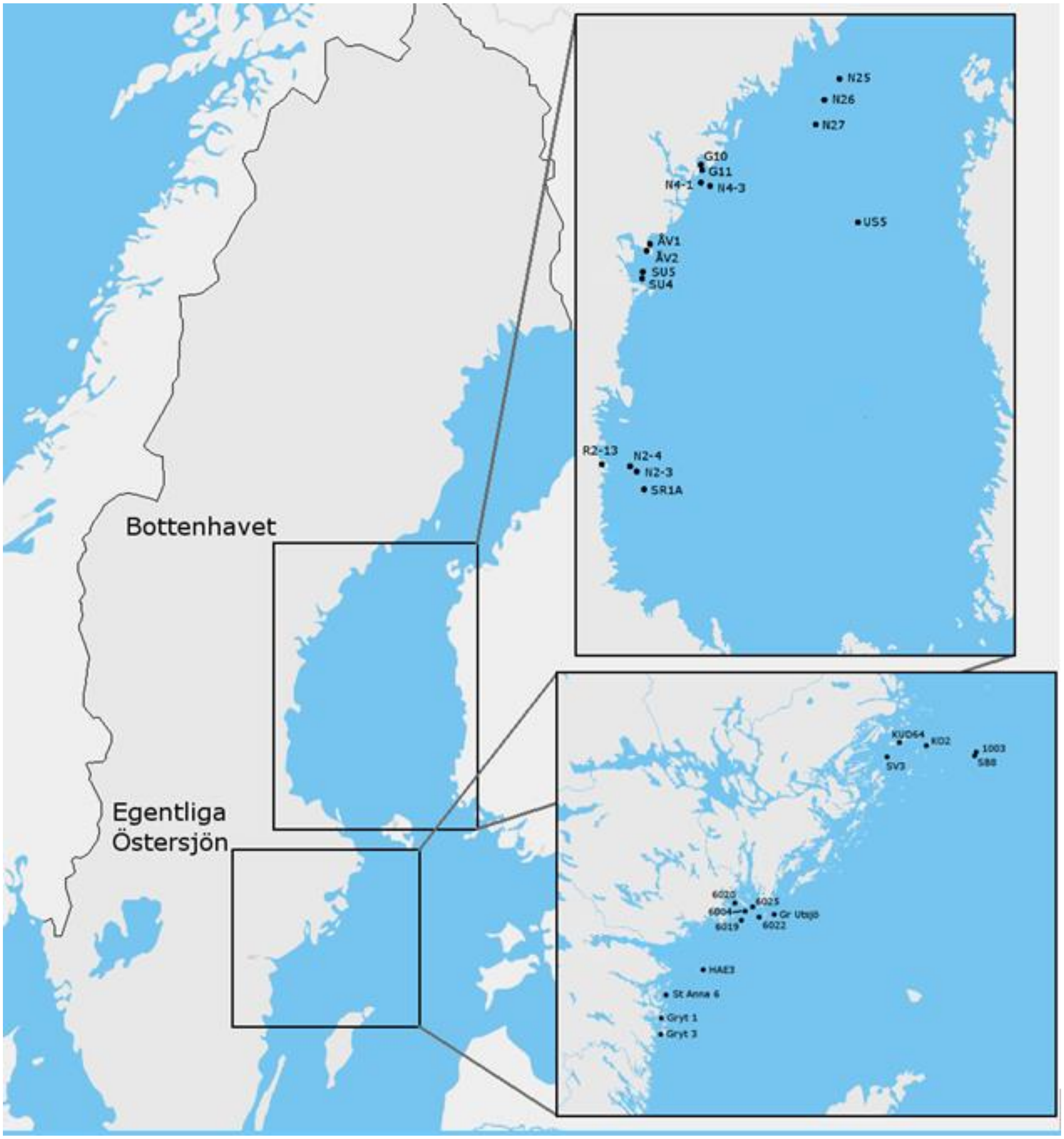

Figure S2. Illustration of nucleoside adduct (M) and its fragmentation pattern. The 2'-deoxyribose and the modification moieties are shown as dR and A, respectively. The protonated dR fragment, with m/z 117.0552, was used for screening of the adducts. The identification of adducts was based on the nucleobase fragment containing A as they are specific to the individual adducts.

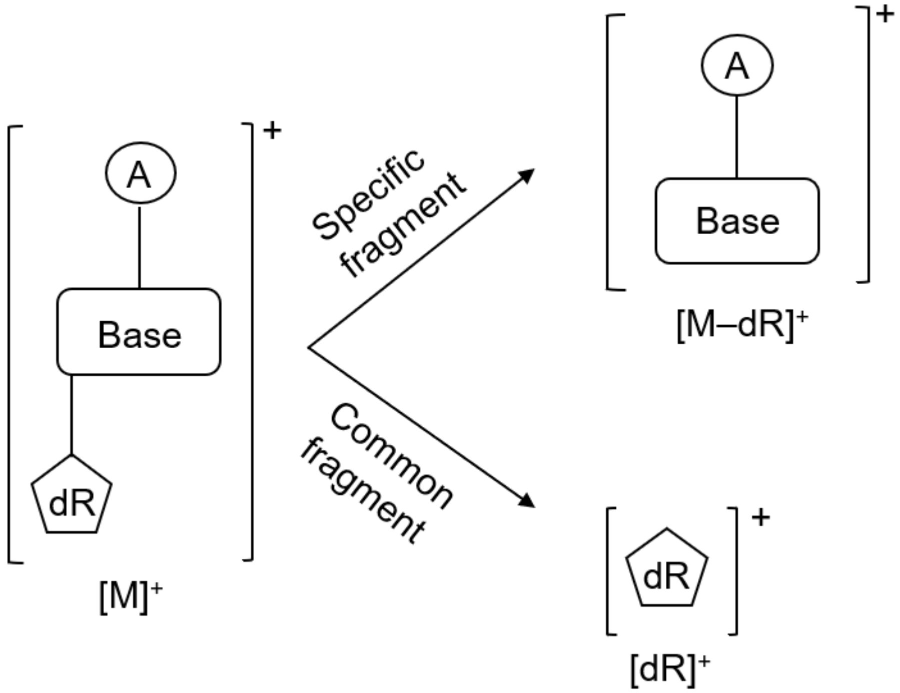

Figure S3. Evaluation of the method for quantification of nucleoside adducts using calf thymus DNA (ctDNA). ctDNA (n=5) was digested and analysed using the same method as that applied for the amphipod samples. The data are shown as normalized mean values and the standard deviations as error bars. For the measured adducts, the method was shown to be repeatable with CV <10%, indicating a low analytical variability.

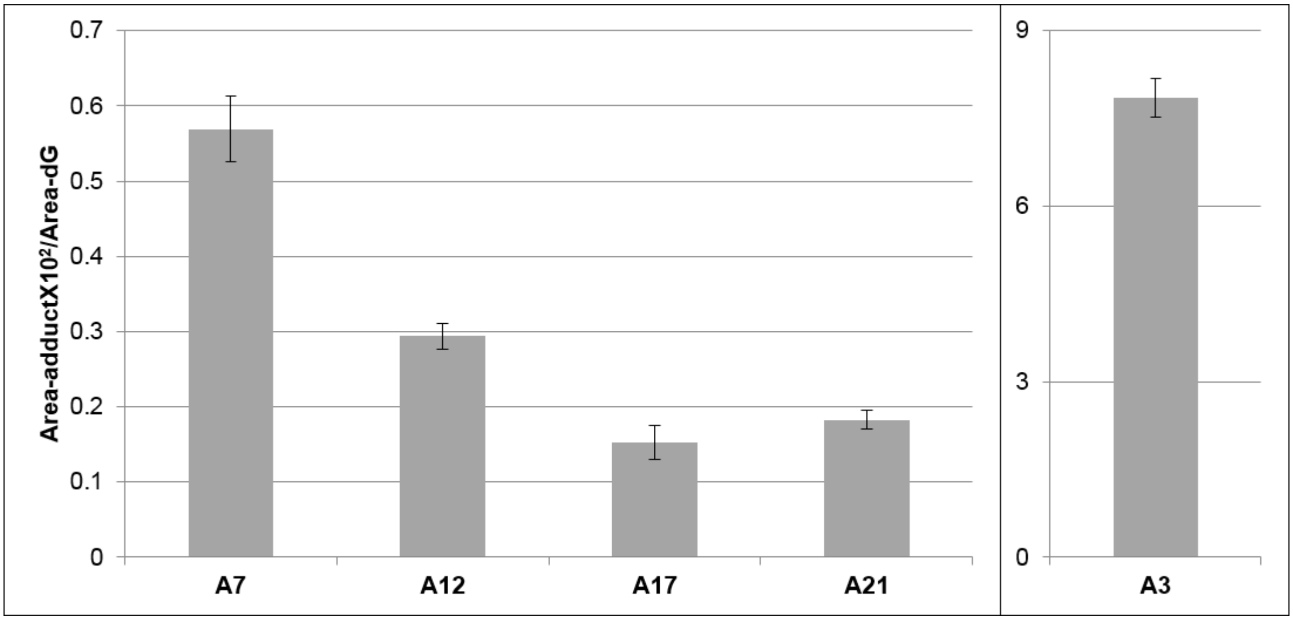

Figure S4. Identification of 5-me-dC (A3), N^6-me-dA (A22) and 8-oxo-dG (A21) in amphipods DNA using respective standards. An overlap of peaks corresponding to the nucleoside adducts in amphipod samples before (continuous curve) and after (dashed curve) spiking of the respective standards using m/z of respective $[M\text{-}dR+H]^+$ confirmed the identification. EIC for A21 in amphipod samples showed two peaks, but only the second peak had a retention time similar to that of the standard, indicating the first peak to be an artefact.

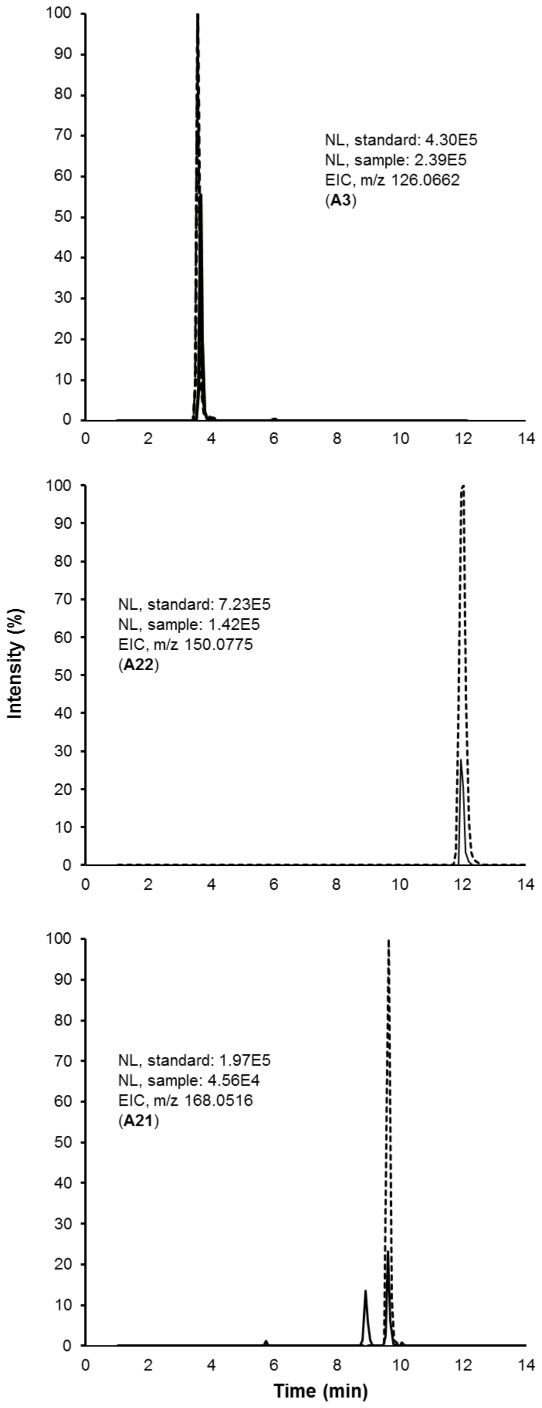

Figure S5. Distribution of the measured nucleoside adducts (as well as dA, dC and T) shown as histograms and box-plots. Peak areas normalized to dG (adduct area × 10^2 / dG area) for (**A**) females and (**B**) embryos are shown on the Y axis. The box represents 25%–75% of the values, with median shown as a horizontal line. The center of the rhombus represents the mean, and its upper and lower edges represent 95% confidence interval for the mean. The whiskers show non-outlier range, and outliers are displayed as dots. Adducts that were below the apparent quantification limit of the instrument (A5 and A10 in females; A1, A5, A10, and A22 in embryos) as well as those identified as sodiated adducts (A4, A13, A16, and A18) are not shown.

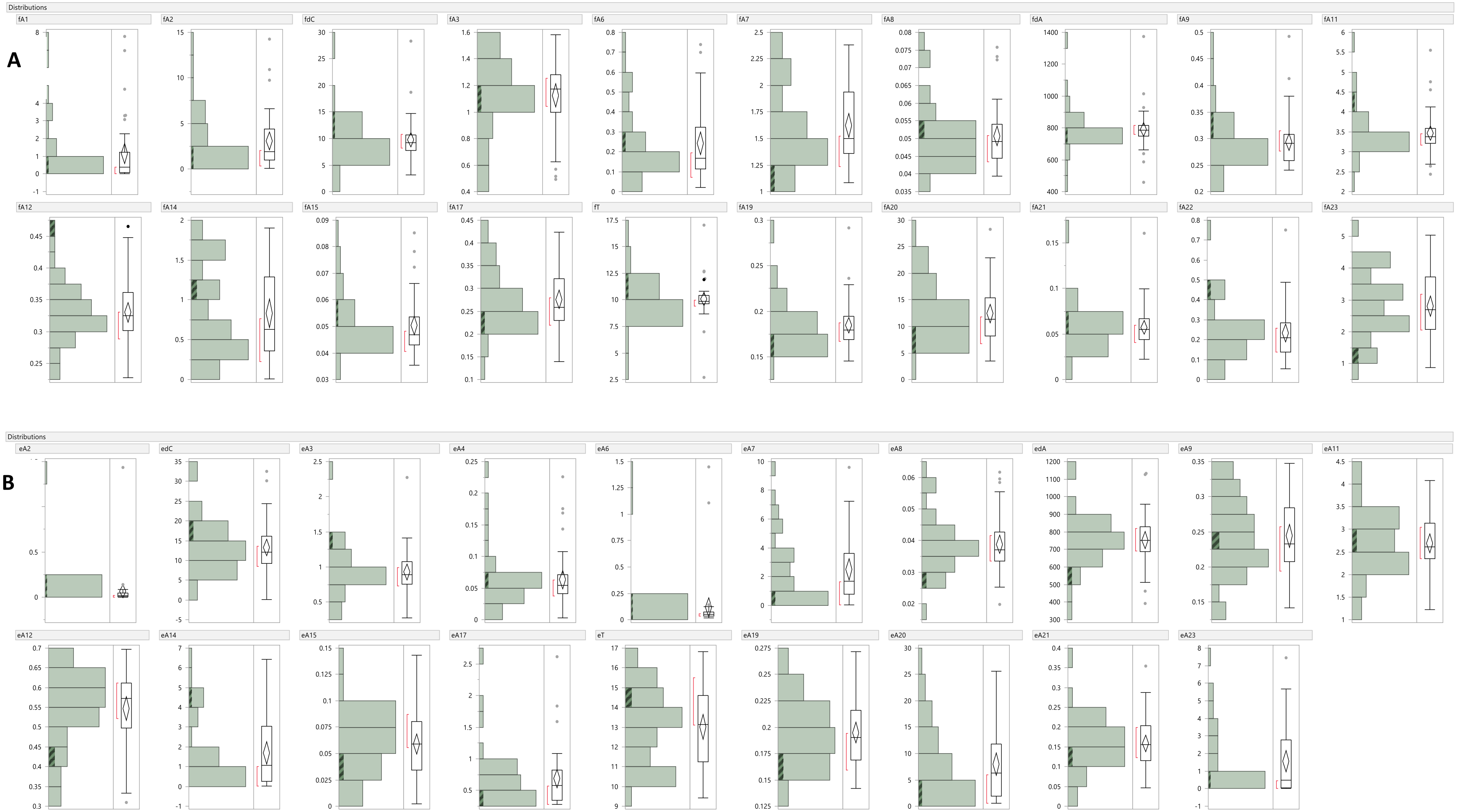

Figure S6. Variations in the normalized peak area for nucleoside adducts as well as dA, dC and T in *M. affinis* females and their embryos. All adduct values are shown on the Y axis as peak areas normalized to dG (adduct area × 10^2 / dG area). The grouping corresponds to embryo (E) and female (F) samples, with healthy (H, left, in blue) and unhealthy (U, right, in red) individuals. In total, 40 gravid females were used generating 40 samples for females and 40 samples for embryos. If >6% of embryos in the brood pouch were malformed, both the female and her embryos were classified as unhealthy (n = 19), otherwise, as healthy (n = 21). Box and whiskers indicate interquartile range and limits for the 95%-confidence interval, respectively; data points above or below the whiskers represent possible outliers. Adducts that were below the apparent quantification limit of the instrument (F: A5 and A10; E: A1, A5, A10 and A22) are not shown.

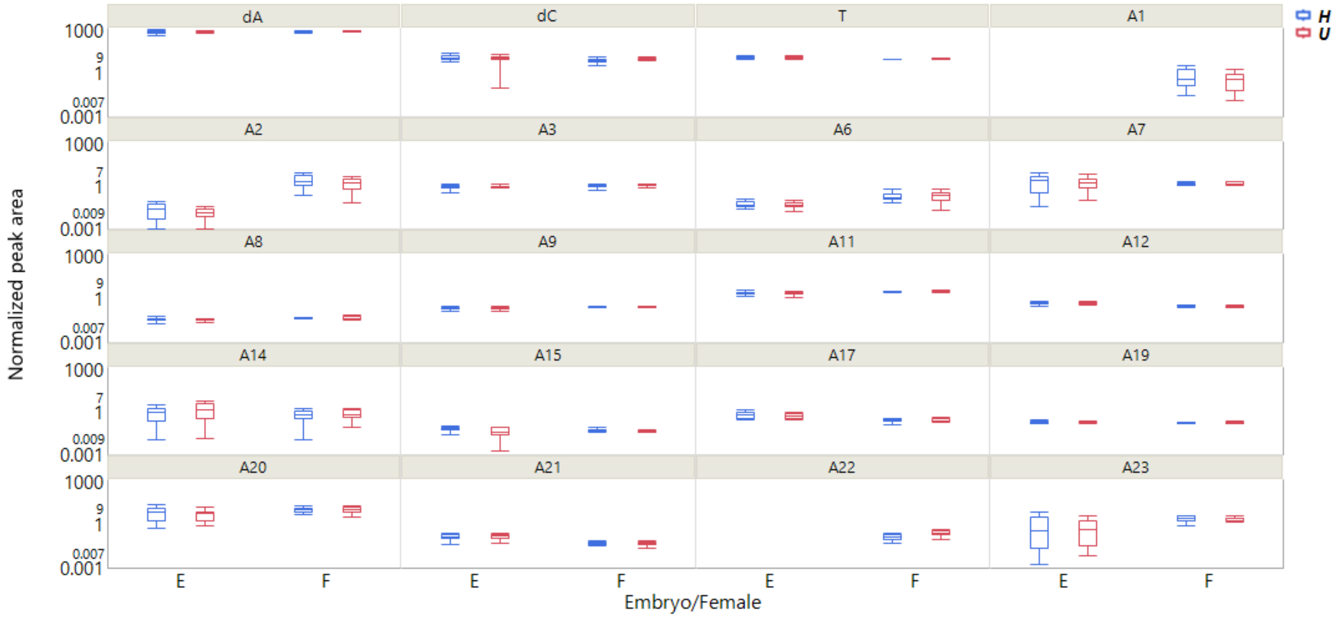

Figure S7. NMDS ordination diagram based on the nucleoside adducts in the females and their embryos with (A) healthy and (B) unhealthy animals. Analysis of group Similarities (ANOSIM) results on the amount of variation in the adduct data attributable to the source (females vs. embryos) are based on Bray-Curtis dissimilarity index. See Fig. 4 for the evaluation of the pooled samples.

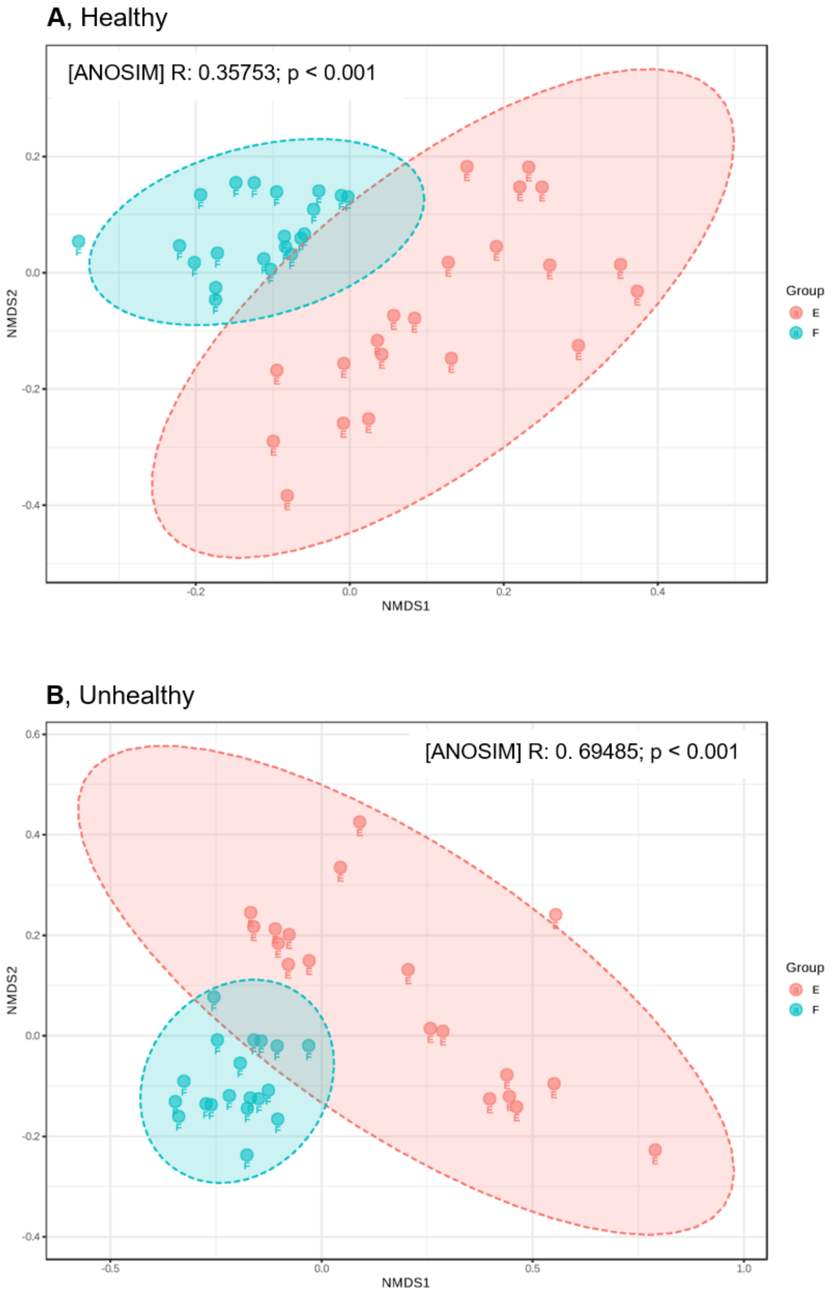

Figure S8. Discrimination of the adductome in healthy and unhealthy amphipods based on area under the receiver operating characteristic (ROC) curve (AUC). For each of the top three discriminating nucleoside adducts, the left panel shows the AUC confidence interval, true positive and false positive rates, and confidence interval (CI), the right panel shows the normalized values for the adducts in healthy and unhealthy individuals. AUC logistic regression approach identified female A22 (AUC = 0.802), A9 (AUC = 0.697), and A3 (AUC = 0.652) to have the greatest specificity and sensitivity for distinguishing the adductome of reproductive pathologies in the amphipods.

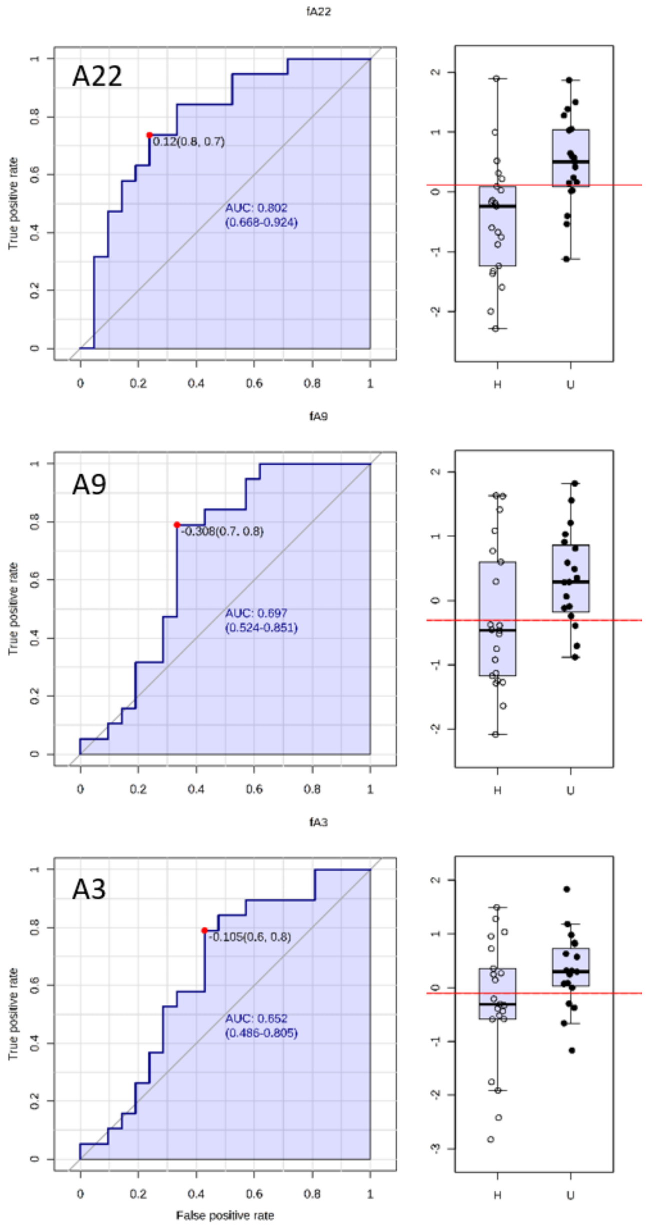

Table S1. Summary of embryo analysis in gravid females. The amphipods were classified as healthy (H1–H21) and unhealthy (U1–U19) based on % embryo aberration.

Amphipod number	Station name	Total eggs	Stage	Malformed	Membrane damage	Arrested development	Dead eggs	Dead brood	% aberration
H1	6004	21	4						0
H2	HAE 3	44	4						0
H3	HAE 3	34	4			1			3
H4	SB8	31	4						0
H5	SB8	35	4						0
H6	6020	32	4						0
H7	KO2	31	6	1					3
H8	1003	29	4			1			3
H9	Gr utsjö	32	6						0
H10	Gr utsjö	32	5						0
H11	KUD 64	52	4						0
H12	St Anna [illegible]	22	6						0
H13	St Anna [illegible]	35	6			1			3
H14	ÅV 2	30	6						0
H15	R2-13	58	6						0
H16	G 10	34	6						0
H17	SU4	41	6						0
H18	ÅV 1	24	5						0
H19	G 11	24	5						0
H20	US 5	19	4						0
H21	SR 1A	21	6			1			5
U1	HAE 3	26	4	2	1	4			27
U2	HAE 3	46	5	4		2			13
U3	KO2	27	6	3					11
U4	KO2	15	4	5					33
U5	KO2	14	4	3	2				36
U6	6020	29	4	6		3			31
U7	KUD 64	25	5	2					8
U8	KUD 64	15	4	1	2				20
U9	KUD 64	13	5	2		1	1		23
U10	St Anna [illegible]	34	6			3	2		9
U11	ÅV 2	23	5	2					9
U12	ÅV 2	26	6	3					12
U13	6020	20	4	2					10
U14	R2-13	55	6	17					31
U15	R2-13	37	8	10		5			41
U16	ÅV 1	23	6	4	1	2	1		30
U17	SU5	29	5	3		1			14
U18	N 27	27	5	3					11
U19	N 25	20	6	1	1			1	10

Table S2. Cross-correlation (Pearson r) for specific nucleoside adducts between the female and the embryo DNA. Adducts with prefix *f* (fA1 to fA23) refer to females, and those marked with *e* (eA2 to eA23) to the embryos; altogether, 40 paired female-brood samples were used. Adducts that were below the apparent quantification limit were excluded. The data were Box-Cox transformed; the significant ($p < 0.05$) correlations are in red. Significant female-embryo correlations for specific adducts (A15, A20 and A23) are in bold.

	Pearson *r*, pair-wise															
Variable	eA2	eA3	eA6	eA7	eA8	eA9	eA11	eA12	eA14	eA15	eA17	eA18	eA19	eA20	eA21	eA23
fA1	0.09	0.24	-0.26	0.13	-0.02	0.24	0.21	0.26	-0.14	0.18	0.05	0.06	0.01	0.00	0.23	0.07
fA2	0.04	0.18	-0.29	-0.11	0.06	0.44	0.41	0.45	-0.47	-0.07	-0.14	-0.13	-0.11	-0.11	0.30	-0.29
fA3	0.06	-0.11	0.30	-0.31	-0.07	-0.25	-0.13	-0.16	-0.01	-0.31	-0.15	-0.11	-0.00	-0.18	-0.07	-0.31
fA6	-0.08	-0.06	0.14	0.36	-0.03	-0.22	0.02	-0.19	0.49	0.25	0.32	0.45	0.27	0.28	0.01	0.47
fA7	0.08	0.01	-0.04	0.22	0.23	-0.19	-0.09	-0.08	0.04	0.16	0.19	-0.02	-0.10	0.23	-0.06	0.30
fA8	0.05	0.01	-0.17	-0.08	0.07	-0.12	0.04	0.02	-0.22	-0.06	-0.03	-0.16	-0.16	0.04	0.07	-0.03
fA9	-0.08	-0.06	0.02	-0.21	0.00	-0.05	0.11	0.08	0.08	-0.21	-0.09	0.07	0.09	-0.17	0.11	-0.24
fA10	0.02	-0.12	-0.01	0.06	0.04	-0.27	-0.08	-0.14	0.15	-0.02	0.04	-0.02	-0.10	0.20	0.01	0.02
fA11	-0.00	-0.00	-0.08	-0.09	0.09	-0.09	-0.04	0.06	-0.01	-0.18	-0.07	-0.02	-0.06	-0.07	-0.07	-0.04
fA12	0.12	-0.01	-0.19	0.10	0.13	-0.22	-0.18	-0.10	-0.01	0.01	0.14	-0.00	-0.07	0.19	-0.16	0.16
fA14	-0.03	-0.19	0.09	-0.26	0.05	-0.10	0.06	-0.03	0.05	-0.28	-0.15	-0.07	-0.05	-0.04	-0.20	-0.23
fA15	0.21	0.09	-0.14	0.32	-0.10	-0.19	-0.19	-0.21	0.09	**0.35**	0.22	0.06	0.02	0.22	-0.00	0.34
fA17	0.02	0.07	-0.20	0.17	-0.08	0.02	0.06	0.02	-0.18	0.04	-0.08	-0.27	-0.30	0.13	0.15	0.09
fA18	0.08	-0.03	-0.20	0.11	-0.04	-0.07	0.02	0.00	-0.12	-0.04	-0.02	-0.16	-0.18	0.13	0.06	0.08
fA19	-0.01	0.03	-0.17	0.08	-0.06	0.01	0.06	0.03	-0.13	-0.07	-0.06	-0.09	-0.09	0.04	0.22	0.02
fA20	0.15	0.03	0.03	0.31	0.30	-0.30	-0.15	-0.14	0.13	0.22	0.32	0.06	0.03	**0.36**	-0.14	0.42
fA21	0.12	-0.01	-0.02	0.41	0.03	-0.38	-0.15	-0.29	0.44	0.17	0.32	0.36	0.18	0.39	-0.05	0.43
fA22	-0.04	-0.30	0.26	-0.11	0.02	-0.23	-0.13	-0.04	0.08	-0.25	0.00	-0.09	0.04	0.07	-0.15	-0.11
fA23	0.10	0.13	-0.12	0.45	0.07	-0.05	-0.05	0.07	0.05	0.28	0.24	-0.00	-0.10	0.27	-0.07	**0.52**

Table S3. Pair-wise correlations (Pearson r) for specific nucleoside adducts within an individual (A) female and (B) embryo DNA. See Table S2 for adduct coding; n = 40 for each group. The data were Box-Cox transformed; the significant ($p < 0.05$) correlations are shown in red. Strong correlations ($r > 0.7$) are indicated in bold.

	(A) Females															
Variable	fA1	fA2	fA3	fA6	fA7	fA8	fA9	fA11	fA12	fA14	fA15	fA17	fA19	fA20	fA21	fA22
fA2	0.42															
fA3	-0.54	-0.14														
fA6	0.17	-0.20	-0.28													
fA7	0.16	0.11	-0.19	0.27												
fA8	-0.06	0.19	0.32	0.14	0.44											
fA9	-0.15	-0.01	0.59	0.28	0.18	0.65										
fA11	-0.10	0.01	0.49	0.22	0.32	**0.73**	**0.88**									
fA12	0.01	-0.05	0.18	0.20	0.18	0.55	0.55	0.69								
fA14	-0.33	-0.19	0.33	0.13	-0.15	-0.04	0.27	0.20	0.06							
fA15	0.18	-0.03	-0.17	0.25	0.13	0.44	0.18	0.25	0.48	-0.21						
fA17	0.04	0.15	-0.11	-0.09	0.27	0.47	0.02	0.08	0.14	-0.28	**0.71**					
fA19	0.04	0.14	0.13	0.15	0.30	**0.73**	0.51	0.51	0.37	-0.18	**0.72**	**0.79**				
fA20	0.18	0.00	-0.28	0.30	**0.86**	0.24	-0.03	0.14	0.13	-0.10	-0.07	0.01	-0.01			
fA21	-0.11	-0.34	0.12	0.53	0.21	0.34	0.52	0.46	0.49	0.27	0.55	0.23	0.46	0.03		
fA22	-0.28	-0.11	0.39	0.03	0.21	0.42	0.57	0.51	0.37	0.27	-0.06	-0.02	0.25	0.16	0.28	
fA23	0.29	0.12	-0.36	0.13	0.66	0.27	-0.17	0.03	0.10	-0.37	0.34	0.54	0.32	0.60	0.08	-0.08

	(B) Embryos													
Variable	eA2	eA3	eA6	eA7	eA8	eA9	eA11	eA12	eA14	eA15	eA17	eA19	eA20	eA21
eA3	0.55													
eA6	0.02	-0.39												
eA7	0.36	0.03	-0.11											
eA8	0.36	0.00	-0.19	0.33										
eA9	-0.16	0.16	-0.29	-0.22	0.06									
eA11	0.04	0.23	-0.16	-0.13	0.27	**0.75**								
eA12	0.05	0.03	-0.22	0.07	0.43	**0.77**	0.65							
eA14	-0.04	-0.05	0.50	0.00	-0.46	-0.60	-0.41	**-0.70**						
eA15	0.45	0.25	-0.08	0.45	0.12	-0.13	-0.01	-0.11	0.17					
eA17	**0.73**	0.14	0.20	0.59	0.47	-0.33	-0.13	-0.04	0.16	0.58				
eA19	0.32	-0.11	0.15	0.39	0.34	-0.04	0.04	0.04	0.12	0.45	0.64			
eA20	0.27	-0.24	-0.11	**0.74**	0.66	-0.31	-0.11	0.05	-0.19	0.27	0.48	0.36		
eA21	-0.06	0.38	-0.29	-0.18	-0.22	0.59	0.57	0.25	-0.16	-0.05	-0.27	-0.07	-0.37	
eA23	0.37	0.08	-0.11	**0.92**	0.21	-0.33	-0.28	-0.04	0.07	0.42	0.59	0.24	0.59	-0.27

Table S4. Logistic regression output. Classification of cases for unhealthy (U) and healthy (H) females. Odds ratio: 22.67, Log odds ratio: 3.12.

Observed	Predicted - U	Predicted - H	Correct, %
U, 19	16	3	84.2
H, 21	4	17	80.9
